# A Simple Test for Cocaine in Local Anæsthetics

**Published:** 1894-07

**Authors:** H. Carlton Smith

**Affiliations:** Boston


### A SIMPLE TEST FOR COCAINE IN LOCAL ANÆSTHETICS.[1]

BY MR. H. CARLTON SMITH, BOSTON.

Mr. President and Gentlemen of the American Academy of Dental Science,—As my interest and attention have this winter been directed towards the study of your profession, it gives me unusual pleasure to be with you this evening and to have met so many of your number.

I have been asked to write a short paper for your meeting on cocaine, and, if possible, give some simple method for its detection which would be of practical value to busy dentists; a test which could be applied, without extended practice in chemical manipulations, to the various mixtures and preparations constantly brought to our notice by the circulars, type-written letters, and numerous other methods of modern advertising.

Some of these preparations, I believe, are worthy of consideration, and even of our confidence, while the use of many others is not to be thought of for a moment by the careful operator who would avoid all risk of serious ill by injecting into the blood such remedies as hydrochlorate of cocaine in solutions of unknown strength.

Cocaine has its uses, it may be, in certain combinations and in not too strong solutions, a very valuable agent for producing local anæsthesia by hypodermic injections, and many claim that the amount used of these preparations is so small that if they were cocaine solutions no harm could result; but I have here a circular issued by the Dickson Manufacturing Company, of Sharon, Pennsylvania, giving a list of twenty-one persons operated upon by Dr. H. F. Dickson, and the number of teeth extracted at a single sitting by the use of Dickson's anæsthetic. The number is in no case less than ten, and runs all the way to twenty-six. According to an analysis made by Professor S. P. Sadtler, of Philadelphia, Dickson's improved anæsthetic contains 3.9 per cent. anhydrous cocaine chlorate, besides chloral hydrate and carbolic acid. Now, if a preparation of this sort be injected on both sides of the tooth, as is usually directed, and repeated a sufficient number of times for the extraction of twenty-six teeth, it seems as though there might be some

---

[1] Read before the American Academy of Dental Science, Boston, March 7, 1894.

question as to the wisdom of using a four-per-cent. solution of cocaine. This drug, as you are all aware, has an unenviable reputation for causing heart-failure, and many fatalities have resulted from its careless use; hence the necessity, or at least the desirability, of such a test as I have mentioned.

This subject was suggested several months since by Dr. William P. Cooke, and during the winter I have given considerable spare time to its consideration and investigation; and this paper will consist of a simple description of my experiments, leaving out a great deal that would be interesting regarding the discovery, history, and preparation of this undeniably wonderful compound.

A test suitable for your purpose I have never seen published nor heard given in any lecture on materia medica, toxicology, or medical chemistry. Cocaine does not give distinctive color reactions, as do many of the alkaloids. One of the most characteristic qualitative tests for its detection, as given by a prominent lecturer on toxicology, is to touch the suspected solution to the tip of the tongue. If numbness ensues, cocaine is probably present. This is a test obviously unsuited to our present purpose. The general alkaloidal reagents precipitate it; but this teaches us nothing, as the majority of alkaloids act in the same way, giving tests of similar appearance. Sulphuric, nitric, and hydrochloric acids, and ferric chloride all give negative results, and it is these reagents that give the distinguishing color tests for many common alkaloids.

A test has been recently published for cocaine, which consists of adding a drop of weak ferric chloride solution and heating the mixture to boiling. A blood-red color is developed, almost resembling ferric sulphocyanate. This color is accounted for by stating that when boiled with water cocaine decomposes, forming ecgonine, methyl alcohol, and benzoic acid. In the same journal this test is rather severely criticised by one who signs himself L. de K., by claiming that he found that the same color could be just as readily obtained by applying the test without cocaine.

Now, Mr. L. de K. is decidedly at fault. In an article in the *Berichter*, vol. xviii., page 2953, Merck states that he obtains cocaine from benzoylecgonine, so the theory of the above is all right; and, moreover, boiling a solution of ferric salt will not and cannot give a blood-red solution; although, if the solution be neutral in reaction, a brick-red precipitate of ferric hydrate will be formed, but the difference between a brick-red precipitate and a blood-red solution we need not waste time in discussing. Among other tests I tried this one, but did not succeed in obtaining satisfactory results until the mixture had stood over night. While this is simple enough, I should feel a little uncertainty about it.

After many fruitless experiments it occurred to me that inasmuch as most alkaloids give reactions with iron salts, it would perhaps be possible to oxidize or reduce the iron in the presence of cocaine, and during the operation find some distinctive test for the alkaloid.

The following was tried. To a solution of cocaine was added a few drops of ferric chloride; the iron was reduced as usual; then a minute fragment of permanganate of potassium partially reoxidized the iron; and, lastly, the addition of a single drop of stannous chloride produced a white precipitate. This disappeared upon shaking, but one or two more drops of the chloride of tin reproduced it, and this time it was permanent. This practically constitutes my test.

Subsequent experiments proved that the use of the permanganate was unnecessary, and it was omitted. The tin salt was next tried alone, and then ferrous salt was used instead of the ferric, but in neither case were the tests so distinctive or satisfactory as when both salts were used in conjunction.

One-per-cent. solutions were then made up of each of the following, and carefully tested for cocaine by simply adding to two or three cubic centimetres of the solution three or four drops each of chloride of iron and chloride of tin. The one-per-cent. solutions were of cinchonidine, quinine, morphine, atropine, caffein, strychnine, and some others of less frequent occurrence; and then, as usual ingredients of dental anæsthetics, chloral hydrate, carbolic acid, camphor, and menthol were used separately and in mixtures, and all with negative results save one,—viz., strychnine. This alkaloid also forms an insoluble salt with tin in presence of ferric chloride. This fact does not detract from the value of the test for dental use, for strychnine can be easily detected by evaporating a little of the solution to dryness and obtaining the fading purple color with sulphuric acid and bichromate of potash which is not obtained with cocaine; and then strychnine is the last thing which would ever be put into a preparation to produce local anæsthesia.

The character of the precipitate formed in the test is peculiar and worthy of notice. It very closely resembles the precipitate of chloride of silver, separating in curdy lumps not easily broken up by simple agitation of the test-tube. If you will allow me, I will show you what the precipitate looks like. In applying the test, dilute the solution with an equal bulk of water. This separates camphor, menthol, or other substances soluble only in strong alcohol. If such a precipitate occurs it should be removed by filtration and the filtrate boiled to remove excess of alcohol.

1. Dorsenia.
2. Dorsenia without cocaine.
3. Solution of cocaine.

The precipitate is easily soluble in dilute oxalic or sulphurous acids.

I notice in the pamphlet advertising Dorsenia a paragraph which I should consider an emphatic warning against the use of cocaine. This seems to be a peculiar feature of these preparations, for, according to a clipping from the *American Druggist and Pharmaceutical Record* of October 12, 1893, Professor Sadtler has analyzed ten different samples of dental anæsthetics, and in nine found cocaine, as follows:

| | Per cent. |
|---|---|
| Dixon's Improved | 3.90 |
| Arophene | 1.46 |
| Jessop's | 2.63 |
| Dorsenia | 0.20 |
| Weinmann's | 5.68 |
| Obtunder | 1.35 |
| Dental Surprise | 1.46 |
| Barr's | None. |
| Eureka | 3.26 |
| Anæstheto Obtundent | 3.39 |

The percentages given are for anhydrous cocaine hydrochlorate.

The test we have been discussing to-night shows the cocaine in these preparations as far as I have been able to try them. I have also used it on the following samples received from Dr. Cooke: menthene, tonalgia, torpedus, and muraline. These all responded except the muraline, in which no change occurred. To be sure that no combination of other ingredients held the cocaine in solution and prevented its separation, I took equal portions of the muraline in separate test-tubes, and to one added a little one-per-cent. solution of cocaine, and to the other as much distilled water, and then by applying the test the cocaine in the first tube was detected without the least difficulty. There is no cocaine in muraline.

In using the test the following facts should be remembered. Strong alcohol will prevent the formation of the precipitate. So, while the official tincture of chloride of iron can be used, it should be diluted with about four times its bulk of water.

Cinchonidine and morphine can be made to give similar precipitations, but *not* under the conditions of the test, as they require a large amount of stannous chloride solution.

Regarding the composition of the precipitate I have nothing to say, as no work has been done on this line.

The limited amount of time which I have been able to give to the subject has made it impossible to experiment with all of the compounds on the market, or with all the possible mixtures that might be made to serve the purpose of a local anæsthetic; but a great many have been tried, and thus far nothing has been found to interfere with or seriously impair the value of the test.

It is certainly safe to regard with strong suspicion any preparation giving the test for cocaine we have tried this evening.

Any more light on the subject, or suggestion of improvement, or possible error I shall be most happy to receive and investigate.

I thank you for your attention.

